# Investigating the factorial structure and measurement invariance of the parent-reported strengths and difficulties questionnaire at 11 years of age from the UK Millennium Cohort Study

**DOI:** 10.1007/s00787-023-02156-1

**Published:** 2023-02-11

**Authors:** Charis Bridger Staatz, Yvonne Kelly, Rebecca E. Lacey, Rebecca Hardy

**Affiliations:** 1 Social Research Institute, Institute of Education, University College London, London, UK; 2 Department of Epidemiology and Public Health, University College London, 1-19 Torrington Place, London, UK; 3 School of Sport, Exercise and Health Sciences, Loughborough University, Loughborough, UK

**Keywords:** Factor analysis, Internalising symptoms, Externalising symptoms, Invariance, Predictive validity

## Abstract

The strengths and difficulties questionnaire (SDQ) consist of five sub-scales that have been used to measure internalising and externalising symptoms in children, typically by combining sum scores of two sub-scales each, and pro-social behaviours. However, the different possible factorial structures that represent these symptoms have not been formally tested in a nationally representative sample of UK children. In addition, it is necessary to assess whether the SDQ is interpreted similarly across subgroups of the population. Exploratory and confirmatory factor analysis were used to test three competing structures for the parent-reported SDQ collected at age 11, the start of adolescence, in the UK Millennium Cohort Study (*n* = 11,519), and measurement invariance was assessed according to sex and a measure of deprivation of the area in which households lived. Internal consistency using ordinal alpha, internal convergent validity and external discriminant validity using average variance explained (AVE), and predictive validity were assessed. A five-factor model and a model with two second-order factors for internalising and externalising symptoms had better model fit than a three-factor model. For both structures, invariance was demonstrated across sex and area-level deprivation. AVE scores for the five-factor model indicated that peer and emotional problems factors were measuring a similar construct, as were the hyperactivity and conduct factors. In the second-order model, AVE scores indicated internalising and externalising symptoms were distinct constructs. A second-order model with two factors for internalising and externalising symptoms is appropriate for use in a cohort of UK children born in 2001/02, and our finding of invariance across sex and area-level deprivation indicate that the SDQ can be used in analysis investigating differences in symptoms across subgroups of the population.

**Supplementary Information:**

The online version contains supplementary material available at 10.1007/s00787-023-02156-1.

## Background

The strengths and difficulties questionnaire (SDQ) is a behavioural screening questionnaire, designed for individuals aged 4–17 years, that assesses behaviour and mental wellbeing, and can be completed by either the parent, teacher or child [1]. The five sub-scales, each with five items, are: (1) emotional symptoms; (2) conduct problems; (3) hyperactivity/inattention; (4) peer relationship problems; (5) pro-social behaviour. These sub-scales were originally developed through theory and further refined through exploratory factor analysis (EFA) [1]. Traditionally when using the SDQ, the mean scores across each sub-scale has been taken. However, this use of sum scores has been criticised as it assumes, firstly, that items in the scale are pure and contain no error, and secondly that all items are equally important in measuring the latent factor [2]. Previous work has used confirmatory factor analysis (CFA) to test the underlying factor structure of the SDQ in different populations, but findings have been mixed [3], with a number of studies not finding adequate support for the five-factor model [4–6]. For example, there has been a reported lack of unidimensionality of factors [5, 6], presence of cross-loadings, with some items being more closely related to factors from other sub-scales [4, 6], and low loadings of items onto their respective factors (< 0.4) [6, 7]. As a result, there has been exploration of alternative factorial structures [3].

An alternative factorial structure that is supported by both theory and empirical work, is one with two distinct factors for internalising and externalising symptoms that are measured by their respective sub-scales: internalising symptoms measured by emotional symptoms and peer relationship problems; and externalising symptoms measured by conduct problems and hyperactivity/inattention [3]. The SDQ could then be modelled using two different factorial structures: (1) a first-order structure with three-factors, representing internalising symptoms, externalising symptoms and pro-social behaviour; or (2) a second-order structure with internalising and externalising symptoms as second-order factors and pro-social behaviour as a first-order factor. Both set of symptoms are concepts commonly used to understand psychopathology and mental health among children. Internalising symptoms are directed inwards to the individual and are predictive of and related to conditions such as depression and anxiety [8]. Externalising symptoms are directed outward from the individual and considered disruptive. They are characterised by impulsivity, lower self-regulation and worse inhibitory control [9] and associated with conditions such as attention deficit hyperactivity disorder (ADHD) [8].

Both factorial structures described have previously been tested in different populations and samples, such as countries across Europe including the UK, with findings providing mixed evidence in support of both structures [3, 10–13]. Some variation may be due to whether the symptoms are reported by the parent, teacher or child, with varying level of cross-informant consistency previously demonstrated [14]. The second-order factor model was first tested in the British Child and Adolescent Mental Health Surveys by Goodman et al. and was found to be most appropriate for the general population, and similarity of measurements was demonstrated across informants (e.g. parent, teacher or child) [3]. A five-factor model was found to be more appropriate among children when screening for disorders [3]. Based on recommendations from this work, a second-order factor structure has often been adopted for research in general populations.

More recently CFA in the UK Millennium Cohort Study (MCS) for ages 3–7 supported a five-factor model [15]. It is possible that the factor structure may change through different stages of development, and especially over the transition to adolescence. This previous study did not investigate the factor structure when participants were age 11, nor did it test a second-order factorial structure for internalising and externalising symptoms [15]. It therefore remains necessary to validate the parent-reported SDQ in a contemporary cohort of children at the beginning of adolescence in the UK and test the appropriateness of adopting a second-order factor structure, ensuring that items group onto constructs as hypothesised and that the constructs measure what is intended.

It is also necessary to test predictive validity—the ability of the factors to predict related outcomes—such as internalising symptoms to predict depression and externalising symptoms to predict ADHD. Moreover, it is important to be able to make comparisons across subgroups of the population such as by sex, ethnicity and socioeconomic position (SEP) [13, 16–18]. To do this, invariance must be demonstrated to ensure the scale used is interpreted the same way between respondent, so that observed variations in symptoms between the groups reflect true disparities and not just differences in the way the symptoms are reported.

Therefore, we aim to investigate the appropriateness of using the parent-report SDQ in the MCS at age 11, through conducting an EFA followed by CFA testing competing factorial structures. We aim to update the work of Goodman et al., by testing the same factorial structures in a contemporary and nationally representative cohort of children approaching adolescence. Additionally, we aim to test the predictive validity and demonstrate invariance of internalising and externalising across subgroups. In addition to sex, invariance according to the deprivation level of the area in which children lived (a marker of area-level SEP) was considered, as a growing body of research has demonstrated how local environments influence mental health, including among children [19].

## Methods

### Data

The MCS is a multidisciplinary longitudinal study of 18,552 families (18,827 children) born in the UK between 2000 and 2002 and recruited at 9 months of age if eligible for the almost universal child benefits scheme [20]. At age 3, recruitment of 692 new eligible families occurred bringing the total number of children to 19,517 (19,243 families). Seven sweeps of data collection have taken place between ages 9 months and 17 years. This study uses data on the SDQ from when cohort members were 11, when 13,287 families took part, and data on mental health diagnosis at ages 14 and 17, when 11,726 and 10,625 families took part, respectively. The analytic sample is limited to the first cohort member in each family, to ensure independence of observations. The MCS adopted methods of random selection in electoral wards of the UK stratified by “ethnic minority” (England only), “disadvantaged” and “advantaged” [21]. Oversampling took place in the disadvantaged and ethnic minority stratum. Further details on the sampling strategy and sampling weights are provided in Methods S1 (supplementary material).

### Variables

Parent reported SDQ at sweep 5 (age 11) was used. The SDQ is composed of 25 items rated on a 3 points scale of “not true”, “somewhat true”, “certainly true”. The 25 items are divided into 5 sub-scales, that combine to give a total difficulties score (scales 1–4), an internalising problems score (scales 1 and 4) and an externalising problems score (scales 2 and 3). The full list of items and how they group into each sub-scale is shown in Table 1, along with the abbreviated names adopted for each item.

**Table 1:** Items in the Strengths and Difficulties Questionnaire in the Millennium Cohort Study

Sub-Scale 1: Emotional Symptom Scale	Sub-Scale 2: Conduct Problems	Sub-Scale 3: Hyperactivity Scale	Sub-Scale 4: Peer Problems	Sub-Scale 5: Pro-Social Scale^b^
Complains of headaches/stomach aches/sickness (*Complains*)	Often has temper tantrums (*Anger)*	Restless, overactive, cannot stay still for long (*Restless)*	Tends to play alone *(Alone)*	Considerate of others’ feelings *(Considerate)*
Often seems worried (*Worried*)	Generally obedient^a^ (*Obedience)*	Constantly fidgeting *(Fidget)*	Has at least one good friend^a^ *(Friend)*	Shares readily with others *(Shares)*
Often unhappy (*Unhappy*)	Fights with or bullies other children (*Aggression)*	Easily distracted *(Attention)*	Generally liked by other children^a^ *(Liked)*	Helpful if someone is hurt, upset or ill *(Helpful)*
Nervous or clingy in new situation (*Anxiety)*	Lies or Cheats *(Lies)*	Can stop and think before acting^a^ *(Impulse)*	Picked on or bullied by other children (*Bullied)*	Kind to younger children *(Kind)*
Many fears, easily scared (*Fear)*	Steals from home, school, elsewhere (*Steals)*	Sees tasks through to the end^a^ *(Task)*	Gets on better with adults *(Adults)*	Often volunteers to help others *(Volunteers)*

Names in brackets indicates abbreviated name used for each question

^a^Indicates positively worded items, that items were reversed when combining to create sub-scales

^b^All items on the Pro-Social Scale are positively worded, so no items in the pro-social scale were reversed

Invariance was tested according to sex of the child, and area deprivation linked to the cohort members address at interview at sweep 5, therefore relating to both the parent and child. Sex of the child was reported by the main respondent at sweep 1 as either “male” or “female”, or at sweep 2 for those that joined the cohort later. The index of multiple deprivation (IMD) was the measure of area-level deprivation used (Further details on the IMD in Methods S2). The IMD was grouped for the present analysis into high deprivation (those in the 30% most deprived areas), low deprivation (those in the 30% least deprived areas) and medium deprivation (the remaining 40%).

A description of the variables related to the main respondent, used to describe characteristics of those who completed the parent-reported SDQ are provided in Supplementary Material (Methods S3 and Table S1).

To explore predictive validity, depression, ADHD and autism/Asperger’s diagnosis at later sweeps were used. At age 17, cohort members were asked if they had ever received a diagnosis of depression (either “yes” or “no”) and the age at which they were diagnosed. These questions were combined retaining those who received a diagnosis aged 13 and older. At age 14, parents were asked if the cohort member has a diagnosis of ADHD and autism/Asperger’s (either “yes” or “no”). Ethnicity of the child, used as a control variable, was reported by the main respondent for the cohort member at sweep 1 or sweep 2. Ethnicity was categorised according to the 2001 UK census categories, and six groups are used: (1) White; (2) Mixed; (3) Indian; (4) Pakistani and Bangladeshi; (5) Black and Black British; (6) Other Ethnic Group.

### Analytic approach

All data cleaning and descriptive analyses were conducted in STATA 15.1 [22], whilst EFA and CFA were conducted in Mplus Version 8.5 [23]. The dataset was split randomly in half, with one half used for developing (EFA) and the other for testing models (CFA). An EFA was conducted before CFA, and is reported on in Supplementary Material. Thresholds used for good fit in both EFA and CFA are TLI/CFI > 0.95; RMSEA < 0.06; SRMR < 0.08 and those considered acceptable are TLI/CFI > 0.9; RMSEA < 0.08 [24].

In the CFA, six different models were compared. Models 1, 3, and 5 were, respectively: a first-order model with five-factors; a first-order three-factor model; a second-order model with five first-order factors and two second-order factors (internalising and externalising symptoms) (Figure 7–2). Models 2, 4 and 6 were the same models but with correlated errors included (Methods S4), identified by the modindices function in Mplus, and by the similarity of construct measured [25]. Correlations were only allowed between errors of items that were measuring the same factor. The same correlated errors were selected for models 2, 4 and 6. The number of correlated errors was limited to prevent the model from becoming saturated. The same 6 models were tested again in a sensitivity analysis (Models 7–12), but with removal of variables that loaded strongly onto more than one factor, highlighted by the EFA.

Observed SDQ variables were ordinal, so the CFA was estimated using the Weighted Least Squares, Mean and Variance Adjusted (WLSMV) estimator, and a polychoric correlation matrix with probit regression was adopted. In CFA, factor loadings greater than 0.5 were deemed acceptable and > 0.7 deemed strong [26]. Ordinal alpha and McDonalds Omega were calculated for each of the factors to demonstrate internal consistency. Average Variance Extracted (AVE) scores were calculated for each factor in the model to assess internal convergent validity and compared to their respective squared correlations to assess external discriminant validity.

Configural, metric and scalar invariance was tested between boys and girls and between levels of area deprivation (i.e. high, medium and low). Invariance was tested using the inbuilt function in Mplus for first-order models. Differences between nested models less than 0.010 for CFI/TLI, 0.015 for RMSEA and 0.030 for SRMR were sought to demonstrate invariance. For the second-order factor, a “top down” approach was adopted where scalar invariance was achieved by demonstrating good fit when constraining intercepts and variances to be equal in the second-order model, and the intercepts of the first-order factors were fixed to zero.

**Fig. 1:**
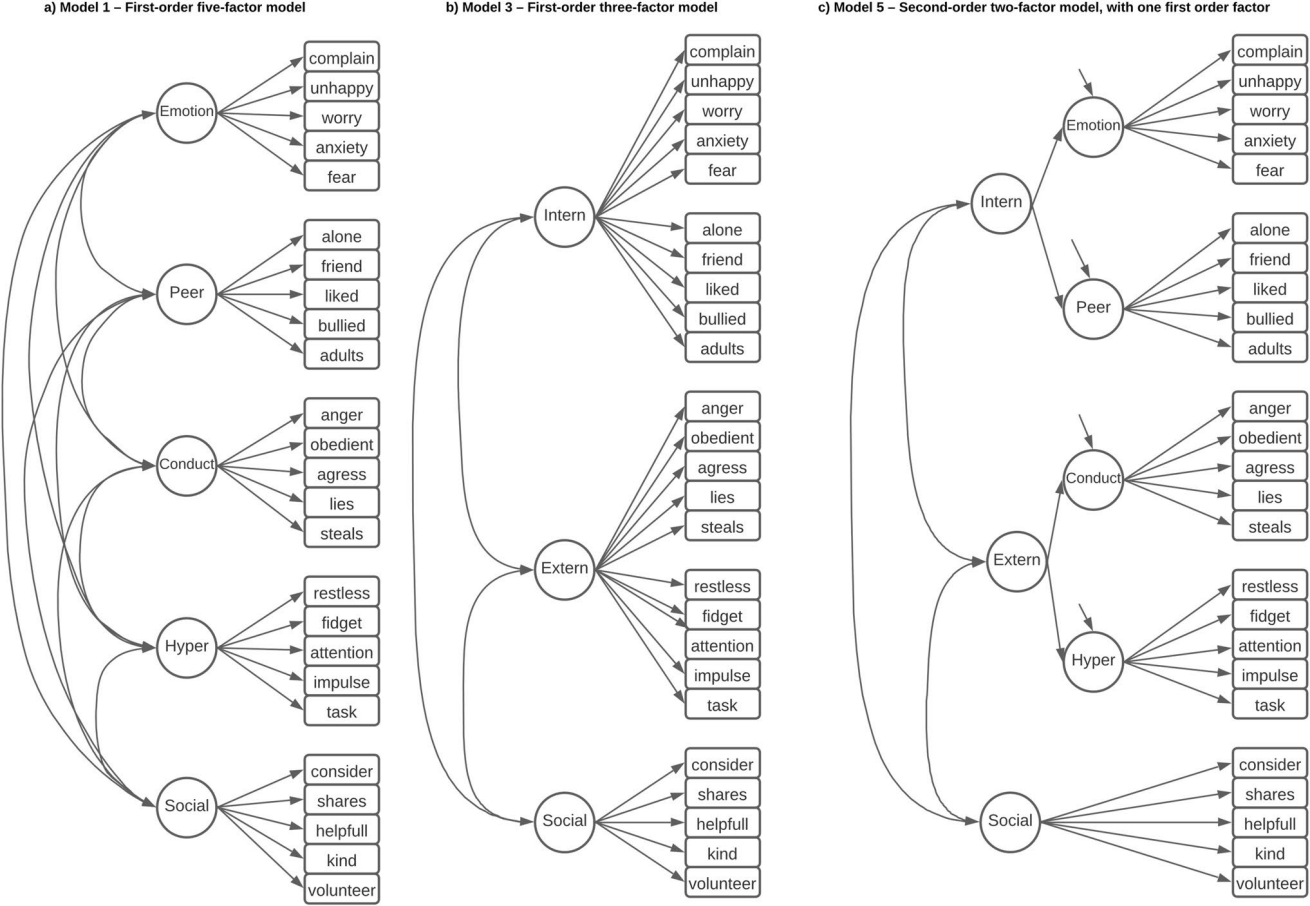
Competing factorial structures. Figure showing competing models tested. Image **a** model 1, a first-order model with five-factors for emotional symptoms, conduct problems, hyperactivity, peer relationship problems and pro-social behaviour. **b** model 3, a first-order model with three-factors for internalising and externalising symptoms and pro-social behaviour. **c** model 5, a second-order model with two-factors from internalising and externalising symptoms, indicated by the first-order factors of emotional symptoms and peer problems for internalising symptoms, and conduct problems and hyperactivity for externalising symptoms, with a separate first-order factor for pro-social behaviour. Models 2, 4 and 6 are the same as 1, 3 and 5, but with correlated errors between some items

Predictive ability of the factors was assessed using probit regression testing associations between the factors from the CFA and each of the diagnostic criteria in an unadjusted model that included all the factors simultaneously. Probit regression coefficients range from − 1 to 1, and are interpreted as the change in the predicted probability given a 1 unit increase in the predictor, with positive values indicating an increased predicted probability. An adjusted model including covariates sex, ethnicity and stratification characteristics was also tested (Fig. 1).

### Analytic sample and missing data

Among those who had completed or partially completed the SDQ (*n* = 12,819), the majority of items had less than 1% missing data and therefore the analysis was limited to complete cases. A total of 11,519 participants were included (Figure S1), with 5,819 (50.5%) in the development dataset and 5700 (49.5%) in the testing dataset. For sensitivity analysis and predictive validity analysis sample sizes differed (Figure S2 and Methods S5).

## Results

### Descriptive results

Characteristics of parents are shown in supplementary Table S2. The respondent who completed the parent-report SDQ was typically the natural parent (99%), as opposed to an adoptive or foster parent, and most often the natural mother (95%). Half were aged 40–49 (51%) and the majority were in work (68%). There were 32% who had a national vocational qualification (NVQ) level 4 (equivalent to a first degree i.e. undergraduate) and 25% with a NVQ level 2, (equivalent to O level/GCSE grades A–C) (further details on equivalent NVQ levels is provided in supplementary table S1). Of the 45% (*n* = 5784) of main respondents who answered the question on their own current depression, 12% reported being currently treated for depression or anxiety.

Distribution for item response by sex for the SDQ is shown in Tables S3-S5. Items that measure internalising symptoms were skewed to indicate fewer internalising traits, whilst pro-social items were skewed to indicate more pro-social behaviour. Items measuring externalising symptoms were typically skewed to indicate fewer externalising traits, but there were three items in boys and two items in girls from the hyperactivity scale that were not skewed. Parents of boys were less likely to report emotional problems than parents of girls, whilst parents of girls typically indicated more pro-social behaviour than parents of boys.

### Exploratory factor analysis and confirmatory factor analysis

In EFA a five-factor structure demonstrated the best model fit, whilst also retaining an eigenvalue greater than one in the EFA (Table S6). Cross-loadings were identified for a number of items in the EFA (See Results S1 and Table S7) and were used to determine removal of items in sensitivity analysis for the CFA.

Fit statistics in CFA for the five-factor model (model 1) and the second-order model (model 5) were comparable and indicated better fit than the three-factor model (model 3, Table 2). Inclusion of correlated errors improved model fit in all cases, therefore, models 2 and 6 were selected for subsequent validity analyses.

**Table 2:** Goodness of fit indices for competing models in confirmatory factor analysis

Model	*χ*^2^	*df*	CFI	TLI	RMSEA	SRMR
1. Baseline five-factor model	2227.962	265	0.921	0.911	0.036	0.062
2. Five-factor model with correlated errors	1710.710	255	0.942	0.931	0.032	0.057
3. Baseline three-factor Model	2947.547	272	0.893	0.882	0.042	0.075
4. Three-factor model with correlated errors	1921.125	262	0.934	0.924	0.033	0.062
5. Second-order two-factor model	2247.816	268	0.921	0.911	0.036	0.064
6. Second-order two-factor model with correlated errors	1741.387	258	0.941	0.931	0.032	0.058

*χ*^*2*^ chi-squared, *df* degrees of freedom, *CFI* comparative fit index, *TLI* Tucker–Lewis index, *RMSEA* root mean square error of approximation, *SRMR* standardized root mean squared residual

**Fig. 2:**
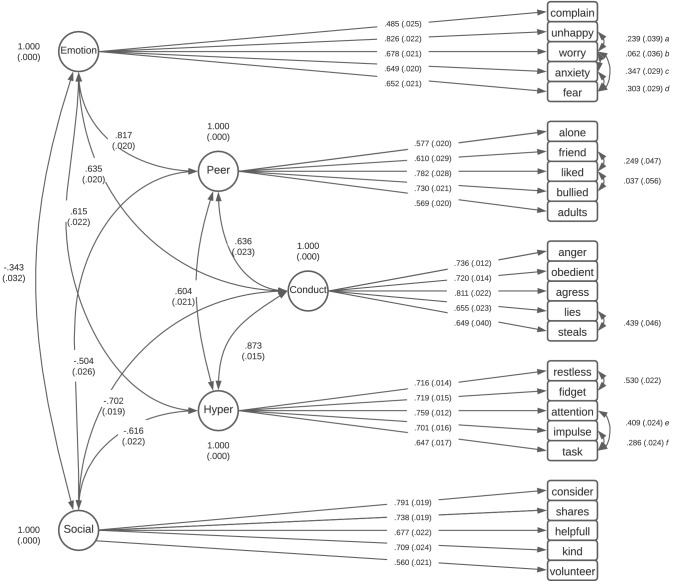
First-order five-factor structures with standardised factor loadings and standard errors. Values in brackets are standard errors (SE). Correlated errors a–f represent **a** unhappy with worry; **b** worry with anxiety; **c** worry with fear; **d** anxiety with fear; **e** attention with task; f impulse with task

**Fig. 3:**
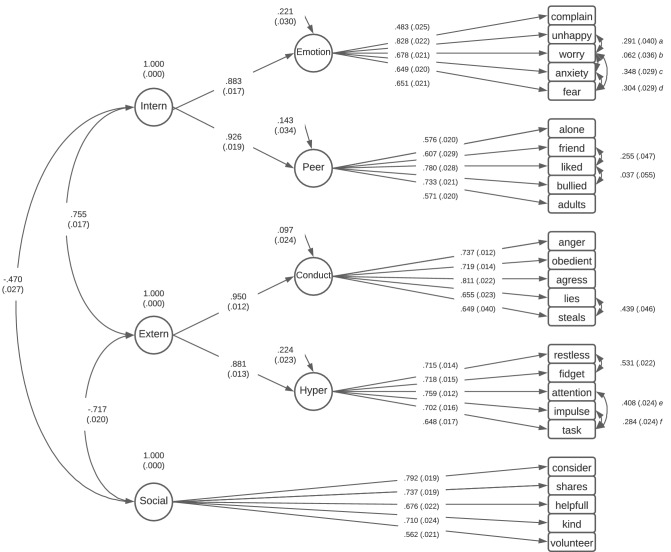
Second-order factor structure with standardised factor loadings and standard errors. Values in brackets are standard errors (SE). Correlated errors **a**–**f** represent **a** unhappy with worry; **b** worry with anxiety; **c** worry with fear; **d** anxiety with fear; **e** attention with task; **f** impulse with task

The majority of the standardised factor loadings (Tables S8–S9) for models 2 and 6 demonstrated acceptable loading (> 0.5). Over half of the first-order item loadings (*N* = 13) exceeded 0.7. Loadings for the emotional symptom factor ranged from 0.49 to 0.83, for conduct problems from 0.65 to 0.81, for the hyperactive scale from 0.65 to 0.76, for the peer problem scale from 0.58 to 0.78, and for the pro-social scale from 0.56 to 0.79 (Fig. 2). Loadings onto the second-order factor were 0.88 and 0.93 for internalising symptoms, and 0.95 and 0.88 for externalising symptoms (Fig. 3). For all items except one (“complains”), the underlying factor explained > 30% of the variance for the items in both the first-order and second-order models. The internal consistency of the sub-scales and the second-order factors, as indicated by the ordinal alpha and McDonalds omega, were all good (> 0.7) (Table S10).

### Measurement invariance

Configural, metric and scalar invariance were demonstrated for the first-order five-factor model across both sex and index of multiple deprivation (Model 2, Table 3). The differences between the configural and metric models, and scalar and metric models ranged from 0.001 to 0.004 (CFI), 0.004 to 0.008 (TLI), 0.001 to 0.002 (RMSEA) and 0.000 to 0.001 (SRMR). For the second-order factor model (model 6), scalar invariance was demonstrated as the model fit was good or acceptable for all indices across all groups tested when constraining intercepts and variances to be equal in the second-order model, and the intercepts of the first-order factors were fixed to zero (Table 4).

**Table 3:** Fit Indices difference tests to confirm configural, metric and scalar measurement invariance in the five-factor first-order model

Model	∆CFI	∆TLI	∆RMSEA	∆SRMR
*Sex*				
Metric vs configural	0.002	0.004	0.001	0.000
Scalar vs metric	0.001	0.004	0.001	0.001
*Index of multiple deprivation*				
Metric vs configural	0.004	0.008	0.001	0.00
Scalar vs metric	0.002	0.006	0.002	0.001

Difference between nested models greater than 0.010 for CFI/TLI, 0.015 for RMSEA and 0.030 for SRMR indicates non-invariance

*CFI* comparative fit index, *TLI* Tucker–Lewis index, *RMSEA* root mean square error of approximation, *SRMR* standardized root mean squared residual

**Table 4:** Fit Indices for the scalar model for the second-order factor

Model	CFI	TLI	RMSEA	SRMR
Sex	0.946	0.943	0.029	0.065
Index of multiple deprivation	0.946	0.944	0.027	0.067

*CFI* comparative fit index, *TLI* Tucker–Lewis index, *RMSEA* root mean square error of approximation, *SRMR* standardized root mean squared residual

### Average variance extracted

For the five-factor model (model 2), AVE scores were above the 0.5 threshold for the emotional problems and conduct problem factors, borderline for the pro-social factor (AVE 0.49), and lower for the peer problem and hyperactivity (Table S11). For the second-order factor (model 6), AVE scores for the internalising and externalising symptoms were 0.81 and 0.84, respectively (Table 5).

**Table 5:** Average variance extracted and squared correlations for second-order factor model

		Correlation			Squared correlation		
	AVE	Internalising	Externalising	Pro-social	Internalising	Externalising	Pro-Social
Internalising	0.81						
Externalising	0.84	0.76			0.57		
Pro-social	0.49	− 0.47	− 0.72		0.22	0.51	

Average variance explained (AVE) scores are the average *R*^2^ score, and represent the average variance explained by the factor in the items that it is measured by

In the five-factor model (model 2), AVE scores for peer problem and emotional problems latent variables were smaller than their squared correlation (0.72), but larger than any other squared correlation, indicating they may be measuring a similar construct but are distinct from other constructs. This was also the case for conduct and hyperactivity factors. When a second-order model is adopted, the AVE scores of internalising (0.81) and externalising (0.84) symptoms were greater than their squared correlation (0.57), indicating they were measuring separate constructs (Table 5). However, the pro-social AVE score was lower than the squared correlation with the externalising symptoms factor (0.51).

### Predictive validity

For the five-factor model (model 2), emotional problems and conduct problems were positively associated with depression in the models adjusted for sex, ethnicity and stratification characteristics (Table 6). Peer problems and hyperactivity positively predicted ADHD diagnosis, whilst emotional problems negatively predicted diagnosis. For autism, peer problems and hyperactivity positively predicted, whilst conduct problems and pro-social behaviours negatively predicted diagnosis.

**Table 6:** Predictive validity of first-order five-factor model

	Mutually adjusted			Minimally adjusted		
	Estimate	SE	*p* value	Estimate	SE	*p* value
*Depression*						
Emotional symptoms	0.31	0.1	0.002	0.29	0.09	0.001
Peer problems	− 0.03	0.08	0.71	− 0.02	0.07	0.75
Conduct problems	0.36	0.16	0.026	0.25	0.13	0.055
Hyperactivity	− 0.19	0.1	0.063	− 0.10	0.09	0.23
Pro-social	0.14	0.09	0.12	0.06	0.08	0.42
*ADHD*						
Emotional symptoms	− 0.44	0.14	0.002	− 0.39	0.13	0.004
Peer problems	0.43	0.14	0.002	0.42	0.13	0.001
Conduct problems	0.09	0.15	0.574	0.13	0.14	0.36
Hyperactivity	0.67	0.12	< 0.001	0.55	0.1	< 0.001
Pro-social	0.09	0.09	0.36	0.11	0.09	0.20
*Autism/Asperger’s*						
Emotional symptoms	− 0.17	0.13	0.19	− 0.17	0.12	0.18
Peer problems	0.69	0.11	< 0.001	0.70	0.19	< 0.001
Conduct problems	− 0.55	0.19	0.003	− 0.51	0.24	0.035
Hyperactivity	0.66	0.13	< 0.001	0.59	0.21	0.005
Pro-social	− 0.22	0.09	0.014	− 0.2	0.11	0.078

Standardised probit regression coefficients and standard errors (SE). In mutually adjusted models, all factors are included in the model at the same time (Emotional Symptoms, Peer Problems, Conduct Problems, Hyperactivity, Pro-Social). In minimally adjusted models, additional adjustments are made for sex, ethnicity and stratification characteristics

*ADHD* Attention Deficit and Hyperactivity Disorder

For the second-order factors (model 6), a higher level of internalising symptoms were associated with increased likelihood of depression, and a higher level of externalising symptoms were associated with ADHD (Table 7). Pro-social symptoms were also positively associated with a formal diagnosis of ADHD. Internalising symptoms were positively related to autism diagnosis whilst pro-social symptoms were negatively related.

**Table 7:** Predictive validity of second-order factors structure

	Unadjusted			Adjusted		
	Estimate	SE	*p* value	Estimate	SE	*p* value
*Depression*						
Internalising symptoms	0.37	0.09	< 0.001	0.32	0.08	< 0.001
Externalising symptoms	0.05	0.1	0.66	0.07	0.09	0.42
Pro-social	0.1	0.07	0.13	0.05	0.06	0.47
*ADHD*						
Internalising symptoms	− 0.16	0.09	0.071	− 0.09	0.08	0.29
Externalising symptoms	0.92	0.12	< 0.001	0.8	0.11	< 0.001
Pro-social	0.14	0.08	0.083	0.15	0.08	0.04
*Autism/Asperger’s*						
Internalising Symptoms	0.54	0.07	< 0.001	0.58	0.15	< 0.001
Externalising Symptoms	0.11	0.1	0.25	0.05	0.09	0.60
Pro-social	− 0.17	0.07	0.018	− 0.14	0.08	0.07

Standardised probit regression coefficients and standard errors (SE). In unadjusted models, all factors are included in the model simultaneously (internalising symptoms, externalising symptoms, pro-social). In adjusted models, adjustments are made for sex, ethnicity and stratification characteristics

### Sensitivity analysis

Items “impulse” and “liked” were removed in sensitivity analysis (models 7–12), as they represent cross-loadings between conceptually distinct factors. The item “considerate” was not removed due to the lower factor loading on the conduct problem factor (0.36), whilst loading strongly onto the pro-social scale factor (0.52). EFA found the five-factor model with cross-loadings removed to also be best fitting, and geomin rotated factor are shown in Table S12. In the CFA, similar to the main analysis the five-factor model with correlated errors (model 8) and the second-order model with correlated errors (model 12) had the best fit (Table S13).

The AVE scores for the five-factor structure (model 8) were similar to the main analysis, whilst slightly higher for the internalising symptoms (0.85) and marginally lower for externalising symptoms (AVE 0.79) in the second-order model (Tables S14-S15). The AVE score for the pro-social factor (0.49) exceeded the squared correlation with the externalising symptom factor (0.47).

Results for predictive validity with cross-loadings removed (model 8 and 12, Tables S16-S17) were comparable to the results in the main analysis. There was additional evidence that internalising symptoms were negatively associated with ADHD at age 14. In the five-factor model, hyperactivity also negatively predicted depression, and emotional symptoms were negatively associated with autism.

## Discussion

The results indicate that a first-order five-factor or a second-order two-factor model for the SDQ is most appropriate in the UK MCS at age 11. Structural validity was demonstrated through EFA and CFA, and internal consistency was demonstrated by ordinal alpha and McDonalds omega for all sub-scales and factors. There was greater evidence of internal convergent validity and external discriminant validity for the second-order factor, with larger AVE scores and lower squared correlations between constructs. Predictive validity was demonstrated for both models, but the second-order factor has associations more consistent with those hypothesised for ADHD and depression diagnosis. Invariance for both models was achieved across groups, demonstrating that the latent constructs were measured in the same way in males and females, and across levels of area deprivation.

Similar to prior research, there was some evidence of items that cross-loaded between factors [4, 6]. Our work indicates that items “impulse” and “liked” could be removed due to cross-loading in EFA, and improved model fit in the CFA. Removal of cross-loadings was explored in sensitivity analysis but did not improve the AVE scores substantially in the first-order five-factor model, although there was some evidence that the pro-social scale was a distinct factor in the second-order model. Similar model fit and predictive validity were demonstrated with and without inclusion of items “impulse” and “liked”, and factor loadings in the main analysis for both items were strong. We were predominantly interested in the difficulties part of the SDQ, and in particular whether a second-order structure was supported for internalising and externalising symptoms. Future analysis that intends to also use the pro-social scale may first wish to explore whether the pro-social scale should be combined with the externalising symptoms factor, whether cross-loadings should be removed, or whether an alternate factor structure is appropriate.

The results of the present study are consistent with work conducted previously using data from the MCS at ages 3–7, which demonstrated a five-factor model fitted the data better when compared with a three-factor model [15]. Similar to the work by Croft et al. (2015), there was mixed support for internal convergent validity in the five-factor model, as demonstrated by low AVE scores for factors [15], although a greater number of factors met the 0.5 threshold in the current analysis. It has been suggested that it is possible to still use the factors with low AVE scores if the model fit is deemed good, factor loading is strong and there is predictive validity [27].

The current analysis finds better internal convergent validity as indicated by AVE scores in the second-order model, which was not tested in the previous MCS analysis, than the first-order five-factor model. Compared to Croft et al., who demonstrated adequate external discriminant validity for all factors in the five-factor model [15], we found poor discriminant validity between emotional symptoms and peer problems, and between hyperactivity and conduct problems indicating they may be measuring similar constructs at 11 years of age. The relation between AVE scores and squared correlations was improved in the second-order model where discriminant validity was achieved for internalising and externalising symptoms. As the model fit of the three-factor model was poor in the CFA, which is an alternative factorial structure that could be adopted to overcome the poor discriminant validity in the five-factor model, there is further justification for adopting a second-order model which had good fit in the CFA and demonstrated discriminant validity.

Croft et al. (2015) demonstrated poor predictive validity for personal, social, and emotional development (PSE) at age 5 by the peer problems and emotional sub-scales of the five-factor model at age 3 in MCS [15]. However, hyperactivity and conduct problems positively predicted ADHD at age 5, and hyperactivity negatively predicted PSE at the same age [15]. In contrast, our analysis finds predictive validity consistent with that hypothesised for the second-order factors for ADHD at 14 and depression at age 13–17. However, autism/Asperger’s was predicted by internalising symptoms and the pro-social scale, but not externalising symptoms indicating less consistent predictive validity. Croft et al. (2015) noted that the SDQ may better predict future internalising rather than externalising problems, such as depression, which had not been collected at the time they carried out their analysis. We were able to demonstrate that this was the case at age 11. This highlights the value of looking at predictive ability of the SDQ measured at different ages, as changes in the factor structure at different stages of development may also impact the association with later diagnosis.

The present study was consistent with work conducted in other populations, which demonstrated a three-factor model with internalising, externalising and a pro-social factor fit the data worse than a five-factor or second-order model [3, 10, 13, 15, 28]. In the UK, similar to our findings, Goodman et al. [3] found a second-order factor model to be the best fit in the general population aged 5–16, but recommended use of a five-factor model in clinical populations when screening for disorders. Support for the second-order structure has been found in an Italian population of children aged 3–15 [12] and in a Danish population of children aged 5–7 and 10–12 which, similar to this study, found it comparable to a five-factor model [11]. However, in Spain, data from children aged 4–14 provided evidence that a five-factor model was a better fit than a second-order model [13]. Similarly, in a Spanish population of adolescents aged 11–19, a five-factor model, or a bi-factorial model was shown to be the most appropriate whilst the second-order factor showed poorer fit [10]. In a cross-country comparison in Europe of adolescents aged 12–17, the five-factor model had the best fit across all countries included, with better fit than the second-order model [28].

We also demonstrate that the first-order five-factor structure is invariant across sex and level of area deprivation. This is similar to work that has found the five-factor model to be invariant across sex, race/ethnicity and income groups among adolescents in the US [17] and across parental education level among a Spanish sample [13], Dutch sample [18], and children with low literacy skills in the US [16]. To the best of our knowledge, no other study has additionally demonstrated invariance for the second-order factor across sex or area-level deprivation suggesting that it is appropriate to look at variation in symptoms across categories of these factors.

## Strengths and limitations

This analysis used a large nationally representative contemporary cohort of children, born in the years 2000/02, on the verge of adolescents in the UK. It was possible to test predictive validity as clinical diagnoses of relevant outcomes were available at later sweeps. However, clinical diagnosis was self-reported by either the parents or cohort members, and there may have been measurement error in the outcome. It is likely the true prevalence of depression, ADHD and Autism/Asperger’s is underestimated as it is probable there are children with these disorders who do not have a clinical diagnosis.

It was not possible to assess measurement invariance using the standard “bottom up” approach for the second-order factor. However, it was possible to demonstrate this for the first-order five-factor structure, which is a pre-condition to demonstrating invariance for second-order factors [29], and scalar invariance was demonstrated for the second-order structure using a “top-down” approach. Therefore, there is reasonable certainty that metric and configural invariance was also achieved for the second-order model, as the scalar model is the strictest form of invariance.

A limitation of this work is that external convergent validity was not demonstrated as there was no comparison between parent-reported SDQ with other reporters such as teachers or cohort members. Cohort member would have been the preferred informant given research that has demonstrated the utility in self-reported mental health [30]. However, in the MCS self-completed SDQs were not available age 11. Although similarity of measures has been demonstrated across informants previously in a UK sample for the SDQ [3], the validity of the current work would be improved with comparison to alternative informants, and caution is necessary in generalising our findings to an SDQ reported by alternative informants. We did not consider characteristics of the parents, other than deprivation of area of residence, such as mental health or individual-level SEP that may have influenced how the SDQ was completed.

It is a possibility that findings could be biased due to missing data, as listwise deletion was used. However, missing data for the majority of items was < 1% for those that at least partially completed the SDQ, thus it is unlikely to impact estimates [31].

## Conclusion

We provide support for use of a second-order factor model when adopting the SDQ at age 11 in the UK. The current CFA demonstrated an acceptable fit for a second-order model, along with better internal convergent, external discriminant and predictive validity than the five-factor model. Overall, the results indicate that the parent-report SDQ appropriately measures internalising and externalising symptoms in the MCS at age 11, and that they are comparable across subgroups of the population. Future researchers wanting to adopt internalising and externalising constructs in the analysis of child psychopathology should use a second-order factor model instead of an alternate three-factor model.

### Supplementary Information

Below is the link to the electronic supplementary material.

Supplementary file1 (DOCX 215 KB)

## Data Availability

All data is available through the UK Data Service with an end user license.
