# Supplementary material for: Investigating the factorial structure and measurement invariance of the parent-reported strengths and difficulties questionnaire at 11 years of age from the UK Millennium Cohort Study
Source: Eur Child Adolesc Psychiatry. 2023 Feb 11;33(1):255–66. doi: 10.1007/s00787-023-02156-1 (PMC10806008; doi:10.1007/s00787-023-02156-1)
Supplement: Supplementary file 1 — Supplementary file1 (DOCX 215 KB) [file 787_2023_2156_MOESM1_ESM.docx]

## Supplementary Material

**Methods S1 – Sampling Strategy and Sampling Weights**

The MCS was designed to be both representative of the UK population, and to provide usable data on sub-groups of children [188]. These sub-groups included children from each of the four countries in the UK (England, Scotland, Wales and Northern Ireland), children living in advantaged and disadvantaged circumstances, and children of ethnic minorities. The MCS therefore adopted methods of random selection in areas of the UK stratified by the above criteria [188].

Ideally, stratification of disadvantaged and ethnic minority children would have been done at the individual level, but this information was only available at the electoral ward level. Only England had an “ethnic minority stratum” because of the small number of ethnic minority groups in Wales, Scotland and Northern Ireland, which at the time was equivalent to 1% of the population [187].

The ethnic stratum was determined as those wards where more than 30% of the children living in the ward were an ethnic minority according to the 1991 census. The disadvantaged stratum was determined as those, other than the ethnic minority stratum, that were rated among the 25% poorest wards according to the Child Poverty Index for England and Wales [189]. The advantaged stratum was all other wards not included in the ethnic or disadvantaged stratum.

Because of the clustered stratified design of the MCS, there are sampling weights to account for the unequal probability of selection [204]. These weights are either country specific or UK wide, dependent on the analysis being conducted. Additionally, because the MCS has experienced attrition at each sweep, attrition and non-response weights have been derived. These weights take into account the probability of non-response of participants and are determined individually for each sweep, and again are either UK wide or country specific.

All analyses were weighted using non-response and sampling weights from the age 11 sweep for the whole UK sample, stratification characteristics and the cluster variable. For predictive validity analysis, weighted estimates were obtained using the non-response and sampling weights at age 14 for autism and ADHD, and non-response weights at age 17 for depression.

**Methods S2 – Description of the Index of Multiple Deprivation**

The IMD is a measure of relative deprivation used in England, with similar measures used in the other countries of the UK. The measure splits England up into Lower Layer Super Output Areas (LSOA), small areas or neighbourhoods with an average population size of 1,500 individuals. The ranking of LSOAs occurs across 39 indicators, which are organised into 7 distinct domains: income; employment; health deprivation and disability; education, skills training; crime; barriers to housing and services; living environment [203]. The income and education domains have a higher weighting in the overall deprivation score than the other domains. Ten IMD groups were created using deciles based on rank. Data on IMD was linked to the address at interview and the LSOA. This was done individually by country, using IMD measures following the Office of the Deputy Prime Minister (ODPM) Indices of Deprivation 2004 in England; Welsh Assembly IMD 2005 in Wales; Scottish Assembly IMD 2004 in Scotland; Northern Ireland Statistics and Research Agency (NISRA) Multiple Deprivation Measure 2005 in Northern Ireland [204]. A single IMD variable was created by combining the IMD from each individual country (England, Scotland, Wales and Northern Ireland).

**Methods S3 – Description of variables used to explore parental characteristics of the main respondent of the parent report SDQ**

Variables used to explore characteristics of the parents were relationship between main respondent and child (e.g natural mother or father, step mother or father, foster mother, grandfather or grandmother, other relative, such as a sibling), the age of the main respondent (grouped ages 16-19, 20-29, 30-39, 40-49, 50+), whether the respondent is in work or not, education level measured by the national vocational qualification (NVQ, see below) and whether the respondent is currently being treated for depression and anxiety (yes or no). All measures used to explore parental characteristics were taken at Sweep 5, when the SDQ was collected.

For education level, respondents were asked about their highest academic or vocational qualification, and this was converted to a National Vocational Qualification (NVQ) scale ranging from “NVQ level 1” to “NVQ level 5”, with an additional group for overseas qualifications and no qualifications. A breakdown of the equivalent qualifications according to the NVQ scale is shown in the Table S1.

**Table S1. National Vocation Qualification Levels and Equivalent Academic and Vocational Qualifications**

| **National Vocation Qualification** | **Equivalent Academic Qualification** | **Equivalent Vocational Qualification** |
| --- | --- | --- |
| No Qualification | None of these qualifications | None of these qualifications |
| Overseas Qualification | Other academic qualification (including from overseas) | Other vocational qualification (including from overseas) |
| NVQ level 1 | GCSE grades D-G | NVQ / SVQ / GSVQ level 1 |
| NVQ level 2 | O level / GCSE grades A-C | Trade apprenticeships or NVQ / SVQ / GSVQ level 2 |
| NVQ level 3 | A / AS / S Levels | NVQ / SVQ / GSVQ level 3 |
| NVQ level 4 | First Degree (i.e undergraduate) or Diploma in Higher Education | Professional qualification at degree level or nursing or other medical qualification |
| NVQ level 5 | Higher degree (i.e PhD or Master’s Degree) | N/A |

***Table S1 Footnotes:*** *Description of different academic and vocational qualifications equivalent to NVQ 1-5. Abbreviations: NVQ – National Vocational Qualification; GCSE - General Certificate of Secondary Education; SVQ – Scottish Vocational Qualifications; GSVQ – General Scottish Vocational Qualifications; O Level – Ordinary Level; A / AS / S Level – Advanced / Advanced Subsidiary / Scholarship Levels; PhD – Doctor of Philosophy.*

**Methods S4 – Included Correlated Errors**

In models 2, 4 and 6, the correlated errors adopted were:

- “fear” with “anxiety” and “worry”
- “anxiety” with “worry”
- “worry” with “unhappy”
- “bullied” with “liked”
- “liked” with “friend”
- “task” with “attention” and “impulse”
- “fidget” with “restless”
- “steals” with “lies”

692 New participants at age 3

5,956 unproductive at age 11

18,551 Participants at 9 months

13,287 Participants at 11 years

12,819 Participants with partial response to the parent-reported SDQ age 11

11,519 Participants with complete response to parents reported SDQ age 11

1,300 participants with missing data on one or more parent-reported SDQ item

1,089 participants with data missing on all parent-reported SDQ items

**Figure S1.** Analytic Sample for Exploratory and Confirmatory Factor Analysis

***Figure S1 Footnote*:** sample size in the development dataset was 5,819, whilst the sample size in the testing set was 5,700.

**Figure S2 – Analytic Sample for Predictive Validity Analysis**


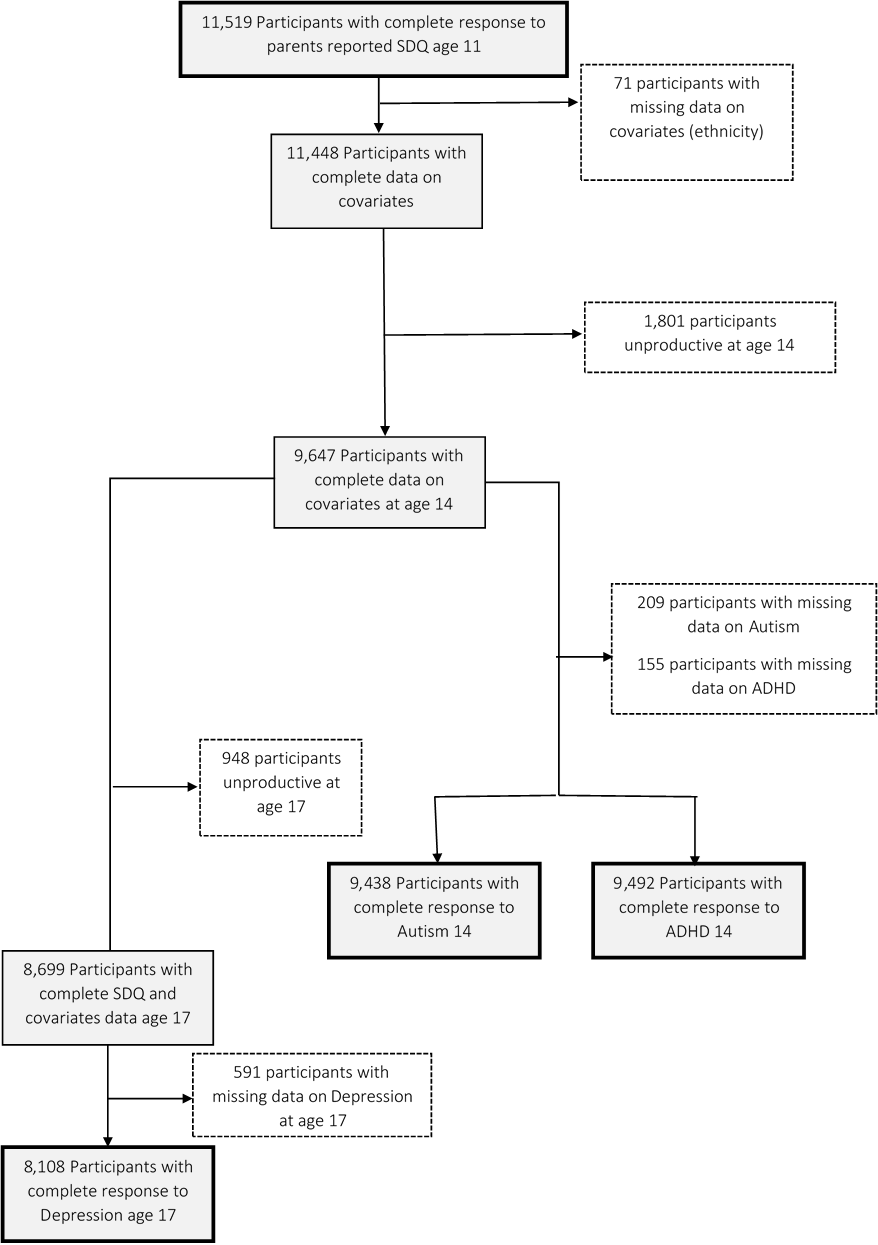


**Methods S5 – Analytic Sample for Sensitivity Analysis**

At age 11, a total of 11,575 participants had complete data on the SDQ for all items apart from “impulse” and “liked” which were removed due to cross-loadings. For sensitivity analysis in EFA and CFA, the sample size for the development side was 5,846 whilst for testing was 5,729. When testing predictive validity in sensitivity analysis, the sample size for depression analysis was 8,032, ADHD analysis was 9,530 and Autism/Asperger’s was 9,474.

**Table S2. Characteristics of respondents who completed the parent reported SDQ at Sweep 5**

| **Parental Characteristics** | **N** | **%** |
| --- | --- | --- |
| **Relation of respondent to Cohort Mother** |  |  |
| Natural parent | 12,710 | 99.1 |
| Of which the mother | 12,237 | 95.5* |
| Adoptive parent | 20 | 0.2 |
| Foster parent | 10 | 0.1 |
| Stepparent, partner of parent | 12 | 0.1 |
| Grandparent | 47 | 0.4 |
| Other relative | 15 | 0.1 |
| Other: sibling/other relative/non-relative | 9 | 0.1 |
| **Age of Respondent** |  |  |
| 16 to 29 | 469 | 3.7 |
| 30 to 39 | 5,123 | 40.0 |
| 40 to 49 | 6,578 | 51.3 |
| 50 plus | 649 | 5.1 |
| **Employment Status of Respondent** |  |  |
| Respondent is in work or on leave | 8,678 | 67.7 |
| Respondent is not in work nor on leave | 4,141 | 32.3 |
| **Education Level of Respondent** |  |  |
| None of these: No Qualification | 1,229 | 9.6 |
| Overseas qual only | 361 | 2.8 |
| NVQ level 1 | 837 | 6.5 |
| NVQ level 2 | 3,165 | 24.7 |
| NVQ level 3 | 1,931 | 15.1 |
| NVQ level 4 | 4,135 | 32.3 |
| NVQ level 5 | 1,125 | 8.8 |
| **Respondent Currently Treated for Depression or Anxiety** |  |  |
| Yes | 1,560 | 12.2 |
| No | 4,224 | 33.0 |

**Table S2 Footnote:** Characteristics are provided for only those individuals who partially or fully responded to the SDQ (N=12,819). * Percentage for mother is given from total respondents (N= 12,819).

**Table S3 – Distribution of responses for items in the internalising symptoms subscales**

|  | **Emotional Scale (Proportion)** | | | **Peer Problems (Proportion)** | | | |
| --- | --- | --- | --- | --- | --- | --- | --- |
|  | ***Boys*** | ***Girls*** | ***Total*** | | ***Boys*** | ***Girls*** | ***Total*** |
|  | **COMPLAINS** | | | | **ALONE** | | |
| **1. Not True** | 0.36 | 0.30 | 0.66 | | 0.34 | 0.35 | 0.69 |
| **2. Somewhat True** | 0.12 | 0.15 | 0.27 | | 0.13 | 0.11 | 0.25 |
| **3. Certainly True** | 0.03 | 0.04 | 0.07 | | 0.04 | 0.02 | 0.06 |
| **Total** | 0.51 | 0.49 | 1.00 | | 0.51 | 0.49 | 1.00 |
| P Value | <0.001 | | | | <0.001 | | |
|  | **WORRIED** | | | | **FRIEND*** | | |
| **1. Not True** | 0.31 | 0.30 | 0.61 | | 0.02 | 0.01 | 0.03 |
| **2. Somewhat True** | 0.16 | 0.16 | 0.32 | | 0.05 | 0.04 | 0.09 |
| **3. Certainly True** | 0.04 | 0.03 | 0.07 | | 0.45 | 0.43 | 0.88 |
| **Total** | 0.51 | 0.49 | 1.00 | | 0.51 | 0.49 | 1.00 |
| P Value | 0.015 | | | | 0.074 | | |
|  | **UNHAPPY** | | | | **LIKED*** | | |
| **1. Not True** | 0.42 | 0.41 | 0.82 | | 0.01 | 0.00 | 0.01 |
| **2. Somewhat True** | 0.08 | 0.07 | 0.15 | | 0.08 | 0.06 | 0.14 |
| **3. Certainly True** | 0.01 | 0.01 | 0.03 | | 0.42 | 0.42 | 0.84 |
| **Total** | 0.51 | 0.49 | 1.00 | | 0.51 | 0.49 | 1.00 |
| P Value | 0.031 | | | | <0.001 | | |
|  | **ANXIETY** | | | | **BULLIED** | | |
| **1. Not True** | 0.31 | 0.29 | 0.60 | | 0.37 | 0.37 | 0.73 |
| **2. Somewhat True** | 0.15 | 0.16 | 0.31 | | 0.12 | 0.10 | 0.21 |
| **3. Certainly True** | 0.05 | 0.04 | 0.08 | | 0.03 | 0.02 | 0.05 |
| **Total** | 0.51 | 0.49 | 1.00 | | 0.51 | 0.49 | 1.00 |
| P Value | 0.015 | | | | 0.005 | | |
|  | **FEAR** | | | | **ADULTS** | | |
| **1. Not True** | 0.36 | 0.33 | 0.69 | | 0.32 | 0.32 | 0.64 |
| **2. Somewhat True** | 0.12 | 0.13 | 0.25 | | 0.15 | 0.13 | 0.28 |
| **3. Certainly True** | 0.03 | 0.03 | 0.06 | | 0.04 | 0.03 | 0.08 |
| **Total** | 0.51 | 0.49 | 1.00 | | 0.51 | 0.49 | 1.00 |
| P Value | 0.17 | | | | 0.012 | | |

***Table S3 Footnote:*** *Table showing proportions for responses to SDQ items that make up internalising symptoms sub-scales, by sex. P-Values are for Pearson’s Chi Squared statistics.*

**Table S4 – Distribution of responses for items in the externalising symptoms subscales**

|  | **Conduct Problems (Proportion)** | | | **Hyperactivity Scale (Proportion)** | | |
| --- | --- | --- | --- | --- | --- | --- |
|  | ***Boys*** | ***Girls*** | ***Total*** | ***Boys*** | ***Girls*** | ***Total*** |
|  | **ANGER** | | | **RESTLESS** | | |
| **1. Not True** | 0.25 | 0.25 | 0.50 | 0.27 | 0.32 | 0.59 |
| **2. Somewhat True** | 0.18 | 0.17 | 0.35 | 0.16 | 0.12 | 0.28 |
| **3. Certainly True** | 0.09 | 0.07 | 0.16 | 0.08 | 0.05 | 0.13 |
| **Total** | 0.51 | 0.49 | 1.00 | 0.51 | 0.49 | 1.00 |
| P Value | <0.001 | | | <0.001 | | |
|  | **OBEDIENCE*** | | | **FIDGET** | | |
| **1. Not True** | 0.03 | 0.02 | 0.05 | 0.32 | 0.36 | 0.69 |
| **2. Somewhat True** | 0.19 | 0.16 | 0.35 | 0.14 | 0.09 | 0.23 |
| **3. Certainly True** | 0.29 | 0.31 | 0.60 | 0.06 | 0.03 | 0.09 |
| **Total** | 0.51 | 0.49 | 1.00 | 0.52 | 0.48 | 1.00 |
| P Value | <0.001 | | | <0.001 | | |
|  | **AGRESSION** | | | **ATTENTION** | | |
| **1. Not True** | 0.46 | 0.46 | 0.91 | 0.19 | 0.25 | 0.44 |
| **2. Somewhat True** | 0.04 | 0.02 | 0.07 | 0.22 | 0.18 | 0.40 |
| **3. Certainly True** | 0.01 | 0.00 | 0.02 | 0.10 | 0.06 | 0.16 |
| **Total** | 0.51 | 0.49 | 1.00 | 0.51 | 0.49 | 1.00 |
| P Value | <0.001 | | | <0.001 | | |
|  | **LIES** | | | **IMPULSE*** | | |
| **1. Not True** | 0.39 | 0.41 | 0.80 | 0.08 | 0.05 | 0.13 |
| **2. Somewhat True** | 0.11 | 0.07 | 0.18 | 0.30 | 0.27 | 0.57 |
| **3. Certainly True** | 0.02 | 0.01 | 0.03 | 0.13 | 0.16 | 0.30 |
| **Total** | 0.51 | 0.49 | 1.00 | 0.51 | 0.49 | 1.00 |
| P Value | <0.001 | | | <0.001 | | |
|  | **STEALS** | | | **TASK*** | | |
| **1. Not True** | 0.49 | 0.47 | 0.97 | 0.09 | 0.05 | 0.13 |
| **2. Somewhat True** | 0.02 | 0.01 | 0.02 | 0.27 | 0.22 | 0.48 |
| **3. Certainly True** | 0.01 | 0.00 | 0.01 | 0.16 | 0.23 | 0.39 |
| **Total** | 0.51 | 0.49 | 1.00 | 0.51 | 0.49 | 1.00 |
| P Value | <0.001 | | | <0.001 | | |

***Table S4 Footnote:*** *Table showing proportions for responses to SDQ items that make up externalising symptoms sub-scales, by sex. P-Values are for Pearson’s Chi Squared statistics.*

|  | **Pro-Social Scale (Proportion)** | | |
| --- | --- | --- | --- |
|  | ***Boys*** | ***Girls*** | ***Total*** |
|  | **CONSIDERATE** | | |
| **1. Not True** | 0.02 | 0.01 | 0.03 |
| **2. Somewhat True** | 0.13 | 0.10 | 0.23 |
| **3. Certainly True** | 0.36 | 0.38 | 0.74 |
| **Total** | 0.51 | 0.49 | 1.00 |
| P Value | <0.001 | | |
|  | **SHARES** | | |
| **1. Not True** | 0.02 | 0.01 | 0.03 |
| **2. Somewhat True** | 0.13 | 0.09 | 0.22 |
| **3. Certainly True** | 0.37 | 0.38 | 0.75 |
| **Total** | 0.52 | 0.48 | 1.00 |
| P Value | <0.001 | | |
|  | **HELPFUL** | | |
| **1. Not True** | 0.01 | 0.01 | 0.02 |
| **2. Somewhat True** | 0.10 | 0.06 | 0.16 |
| **3. Certainly True** | 0.40 | 0.42 | 0.82 |
| **Total** | 0.51 | 0.49 | 1.00 |
| P Value | <0.001 | | |
|  | **KIND** | | |
| **1. Not True** | 0.01 | 0.00 | 0.01 |
| **2. Somewhat True** | 0.08 | 0.04 | 0.11 |
| **3. Certainly True** | 0.43 | 0.44 | 0.88 |
| **Total** | 0.51 | 0.49 | 1.00 |
| P Value | <0.001 | | |
|  | **VOLUNTEERS** | | |
| **1. Not True** | 0.02 | 0.01 | 0.03 |
| **2. Somewhat True** | 0.21 | 0.11 | 0.32 |
| **3. Certainly True** | 0.29 | 0.37 | 0.65 |
| **Total** | 0.51 | 0.49 | 1.00 |
| P Value | <0.001 | | |

**Table S5 – Distribution of responses for items in the pro-social symptoms subscales**

***Table S5 Footnote:*** *Table showing proportions for responses to SDQ items that make up pro-social sub-scale, by sex. P-Values are for Pearson’s Chi Squared statistics.*

**Table S6 - Eigenvalues and Model Fit for Exploratory Factor Analysis (EFA) Models**

| **Model** | **Eigenvalues** | **Degrees of freedom** | **χ2** | **CFI** | **TLI** | **RMSEA** | **SRMR** |
| --- | --- | --- | --- | --- | --- | --- | --- |
| 1- Factor Model | 9.20 | 275 | 6070.33 | 0.799 | 0.781 | 0.060 | 0.110 |
| 2- Factor Model | 2.57 | 251 | 3348.39 | 0.892 | 0.871 | 0.046 | 0.076 |
| 3- Factor Model | 1.71 | 228 | 1910.61 | 0.942 | 0.923 | 0.036 | 0.055 |
| 4- Factor Model | 1.28 | 206 | 1325.04 | 0.961 | 0.943 | 0.031 | 0.044 |
| 5- Factor Model | 1.17 | 185 | 844.40 | 0.977 | 0.963 | 0.025 | 0.034 |
| 6- Factor Model | 0.86 | 165 | 534.34 | 0.987 | 0.977 | 0.020 | 0.027 |
| 7- Factor Model | 0.84 | 146 | 353.74 | 0.993 | 0.985 | 0.016 | 0.020 |
| 8- Factor Model | 0.69 | 128 | 245.02 | 0.996 | 0.990 | 0.013 | 0.016 |

***Table S6 Footnote:*** *Abbreviations: χ2 – chi-squared; CFI – Comparative Fit Index; TLI – Tucker-Lewis Index; RMSEA – Root Mean Square Error of Approximation; SRMR – standardized root mean squared residual.*

**Results S1 – Cross-loadings identified in exploratory factor analysis**

Geomin rotated loadings indicated a unique factor for emotional symptoms, but with a weaker loading for the item “complain” (0.36) (Table S5). There were cross-loadings for the items “liked” (loading onto both peer problem and pro-social scales), “restless” and “fidget” (hyperactivity and conduct scales), and weak cross-loadings for “impulse” (pro-social and the hyperactivity scales), and “considerate” (pro-social and conduct problem scales).

**Table S7 - Geomin Rotated Loadings for Five-Factor Model**

|  | **Factor 1** | **Factor 2** | **Factor 3** | **Factor 4** | **Factor 5** |
| --- | --- | --- | --- | --- | --- |
| **Emotional Symptom Scale** | | | | | |
| Complains | 0.360* | 0.273* | 0.153* | -0.014 | 0.034 |
| Unhappy | 0.623* | 0.311* | 0.011 | -0.064* | 0.128* |
| Worried | 0.767* | 0.056* | 0.056* | -0.005 | 0.035 |
| Anxiety | 0.625* | -0.045 | -0.079* | 0.108* | 0 |
| Fear | 0.798* | -0.009 | 0.004 | 0.055* | -0.011 |
| **Peer Problems** | | | | | |
| Alone | 0.242* | -0.169* | -0.177* | -0.009 | 0.516* |
| Friend | 0.017 | -0.030 | -0.290* | 0.032 | 0.545* |
| Liked | 0.112* | 0.022 | -0.433* | 0.034 | 0.526* |
| Bullied | 0.339* | 0.149* | -0.015 | 0.042 | 0.398* |
| Adults | 0.078 | 0.056 | 0.005 | -0.007 | 0.611* |
| **Hyperactivity Scale** | | | | | |
| Restless | -0.124* | 0.461* | 0.047* | 0.486* | 0.257* |
| Fidget | -0.040* | 0.419* | 0.051* | 0.477* | 0.279* |
| Attention | 0.085* | 0.201* | -0.128* | 0.665* | 0.038 |
| Impulse | 0.021 | 0.206* | -0.365* | 0.396* | -0.045 |
| Task | 0.062* | -0.066 | -0.415* | 0.718* | -0.018 |
| **Conduct Problems** | | | | | |
| Anger | 0.170* | 0.596* | -0.060* | 0.100* | -0.025 |
| Obedience | -0.007 | 0.504* | -0.348* | 0.097* | -0.085* |
| Aggression | 0.099* | 0.552* | -0.198* | 0.015 | 0.194* |
| Lies | 0.140* | 0.641* | -0.085* | 0.054 | -0.080* |
| Steals | 0.05 | 0.778* | -0.115 | -0.091 | -0.003 |
| **Pro-Social Scale** | | | | | |
| Considerate | 0.025 | -0.355* | 0.521* | 0.055* | -0.045 |
| Shares | 0.011 | -0.206* | 0.566* | 0.076* | -0.097* |
| Helpful | 0.055* | -0.041 | 0.750* | 0.091* | -0.042 |
| Kind | 0.055 | -0.110* | 0.665* | 0.002 | -0.044 |
| Volunteers | -0.009 | 0.02 | 0.707* | -0.096* | 0.208* |

***Table S7 Footnote:*** ** indicates p<0.05. Loadings greater or equal to 0.4 are highlighted in dark grey. Greater or equal to 0.35 are highlighted in light grey.*

**Table S8 - Standardised Factor Loadings for the First-Order Five-Factor Model, with Correlated Errors (Model 2).**

| **Items** | **Loadings** | **S/E** | **R^2^** | **P Values** |
| --- | --- | --- | --- | --- |
| **Emotional Symptom Scale** | | | | |
| *Complains* | 0.49 | 0.03 | 0.24 | <0.001 |
| *Unhappy* | 0.83 | 0.02 | 0.68 | <0.001 |
| *Worried* | 0.68 | 0.02 | 0.46 | <0.001 |
| *Anxiety* | 0.65 | 0.02 | 0.42 | <0.001 |
| *Fear* | 0.65 | 0.02 | 0.43 | <0.001 |
| **Conduct Problems** |  |  |  |  |
| *Anger* | 0.74 | 0.01 | 0.51 | <0.001 |
| *Obedience* | 0.72 | 0.01 | 0.52 | <0.001 |
| *Aggression* | 0.81 | 0.02 | 0.58 | <0.001 |
| *Lies* | 0.66 | 0.02 | 0.49 | <0.001 |
| *Steals* | 0.65 | 0.04 | 0.42 | <0.001 |
| **Hyperactivity Scale** |  |  |  |  |
| *Restless* | 0.72 | 0.01 | 0.33 | <0.001 |
| *Fidget* | 0.72 | 0.02 | 0.37 | <0.001 |
| *Attention* | 0.76 | 0.01 | 0.61 | <0.001 |
| *Impulse* | 0.70 | 0.02 | 0.53 | <0.001 |
| *Task* | 0.65 | 0.02 | 0.32 | <0.001 |
| **Peer Problems** |  |  |  |  |
| *Alone* | 0.58 | 0.02 | 0.54 | <0.001 |
| *Friend* | 0.61 | 0.03 | 0.52 | <0.001 |
| *Liked* | 0.78 | 0.03 | 0.66 | <0.001 |
| *Bullied* | 0.73 | 0.02 | 0.43 | <0.001 |
| *Adults* | 0.57 | 0.02 | 0.42 | <0.001 |
| **Pro-Social Scale** |  |  |  |  |
| *Considerate* | 0.79 | 0.02 | 0.63 | <0.001 |
| *Shares* | 0.74 | 0.02 | 0.55 | <0.001 |
| *Helpful* | 0.68 | 0.02 | 0.46 | <0.001 |
| *Kind* | 0.71 | 0.02 | 0.5 | <0.001 |
| *Volunteers* | 0.56 | 0.02 | 0.31 | <0.001 |

**Table S9 - Standardised Factor Loadings for the Second-Order Two-Factor Model, with Correlated Errors (Model 6).**

| **Items** | **Loadings** | **S/E** | **R^2^** | **P Values** |
| --- | --- | --- | --- | --- |
| **First-order factors** | | | | |
| **Emotional Symptom Scale** |  |  |  |  |
| *Complains* | 0.48 | 0.03 | 0.23 | <0.001 |
| *Unhappy* | 0.83 | 0.02 | 0.69 | <0.001 |
| *Worried* | 0.68 | 0.02 | 0.46 | <0.001 |
| *Anxiety* | 0.65 | 0.02 | 0.42 | <0.001 |
| *Fear* | 0.65 | 0.02 | 0.42 | <0.001 |
| **Conduct Problems** |  |  |  |  |
| *Anger* | 0.74 | 0.01 | 0.54 | <0.001 |
| *Obedience* | 0.72 | 0.01 | 0.52 | <0.001 |
| *Aggression* | 0.81 | 0.02 | 0.66 | <0.001 |
| *Lies* | 0.66 | 0.02 | 0.43 | <0.001 |
| *Steals* | 0.65 | 0.04 | 0.42 | <0.001 |
| **Hyperactivity Scale** |  |  |  |  |
| *Restless* | 0.72 | 0.01 | 0.51 | <0.001 |
| *Fidget* | 0.72 | 0.02 | 0.52 | <0.001 |
| *Attention* | 0.76 | 0.01 | 0.58 | <0.001 |
| *Impulse* | 0.70 | 0.02 | 0.49 | <0.001 |
| *Task* | 0.65 | 0.02 | 0.42 | <0.001 |
| **Peer Problems** |  |  |  |  |
| *Alone* | 0.58 | 0.02 | 0.33 | <0.001 |
| *Friend* | 0.61 | 0.03 | 0.37 | <0.001 |
| *Liked* | 0.78 | 0.03 | 0.61 | <0.001 |
| *Bullied* | 0.73 | 0.02 | 0.54 | <0.001 |
| *Adults* | 0.57 | 0.02 | 0.33 | <0.001 |
| **Pro-Social Scale** |  |  |  |  |
| *Considerate* | 0.79 | 0.02 | 0.63 | <0.001 |
| *Shares* | 0.74 | 0.02 | 0.54 | <0.001 |
| *Helpful* | 0.68 | 0.02 | 0.46 | <0.001 |
| *Kind* | 0.71 | 0.02 | 0.50 | <0.001 |
| *Volunteers* | 0.56 | 0.02 | 0.32 | <0.001 |
| **Second-order factors** | | | | |
| **Internalising** |  |  |  |  |
| *Emotional Symptom Scale* | 0.88 | 0.02 | 0.78 | <0.001 |
| *Peer Problems* | 0.93 | 0.02 | 0.86 | <0.001 |
| **Externalising** |  |  |  |  |
| *Conduct Problems* | 0.95 | 0.01 | 0.90 | <0.001 |
| *Hyperactivity Scale* | 0.88 | 0.01 | 0.78 | <0.001 |

**Table S10 - Ordinal Alpha for Strengths and Difficulties Sub-Scales**

|  | **Emotional Symptom Scale** | **Conduct Problems** | **Hyperactivity Scale** | **Peer Problems** | **Pro-Social Scale** | **Internalising**  **Symptoms** | **Externalising**  **Symptoms** |
| --- | --- | --- | --- | --- | --- | --- | --- |
| Average correlation (r): | 0.48 | 0.52 | 0.55 | 0.44 | 0.48 | 0.46 | 0.54 |
| Items in scale (k): | 5 | 5 | 5 | 5 | 5 | 10 | 10 |
| Ordinal Alpha: | 0.82 | 0.84 | 0.86 | 0.8 | 0.82 | 0.89 | 0.92 |
| McDonald’s Omega | 0.83 | 0.85 | 0.80 | 0.99 | 0.85 | 0.93 | 0.87 |

***Table S10 Footnote:*** *Ordinal alpha for items in measurement scales. Ordinal alpha represented by the equation (k*r)/(1+(k-1)*r)*

**Table S11 - Average Variance Extracted and Squared Correlations for Five-Factor Model**

|  | | **Correlation** | | | | | **Squared Correlation** | | | | |
| --- | --- | --- | --- | --- | --- | --- | --- | --- | --- | --- | --- |
|  | **AVE** | ***Peer*** | ***Emotion*** | ***Conduct*** | ***Hyper*** | ***Social*** | ***Peer*** | ***Emotion*** | ***Conduct*** | ***Hyper*** | ***Social*** |
| **Peer** | 0.44 |  |  |  |  |  |  |  |  |  |  |
| **Emotion** | 0.51 | 0.85 |  |  |  |  | 0.72 |  |  |  |  |
| **Conduct** | 0.50 | 0.64 | 0.60 |  |  |  | 0.40 | 0.36 |  |  |  |
| **Hyper** | 0.43 | 0.59 | 0.63 | 0.79 |  |  | 0.35 | 0.40 | 0.62 |  |  |
| **Social** | 0.49 | -0.41 | -0.34 | -0.70 | -0.51 |  | 0.17 | 0.12 | 0.49 | 0.26 |  |

***Table S11:*** *AVE scores are the average R^2^ score, and represent* *the average variance explained by the factor in the items that it is measured by. Shorthand name “Peer” refers to Peer Problems, “Emotion” to Emotional Symptoms, “Conduct” to Conduct Problems, “Hyper” to Hyperactivity and “Social” to Pro-Social. Abbreviation: AVE – Average Variance Explained.*

**Table S12 - geomin rotation loadings factor model without cross-loadings (sensitivity analysis)**

|  | **Factor 1** | **Factor 2** | **Factor 3** | **Factor 4** | **Factor 5** |
| --- | --- | --- | --- | --- | --- |
| **Emotional Symptom Scale** | | | | | |
| Complains | 0.347* | 0.274* | 0.002 | 0.147* | 0.034 |
| Unhappy | 0.640* | 0.359* | -0.067* | 0.013 | 0.084* |
| Worried | 0.777* | 0.102* | -0.016 | 0.064* | -0.014 |
| Anxiety | 0.625* | -0.061* | 0.123* | -0.100* | -0.002 |
| Fear | 0.794* | 0.01 | 0.054* | -0.003 | -0.038 |
| **Conduct Problems** | | | | | |
| Anger | 0.142* | 0.584* | 0.134* | -0.039 | -0.006 |
| Obedience | -0.021 | 0.531* | 0.125* | -0.296* | -0.106* |
| Aggression | 0.115* | 0.541* | 0.04 | -0.190* | 0.207* |
| Lies | 0.101* | 0.648* | 0.086* | -0.057 | -0.059 |
| Steals | 0.013 | 0.801* | -0.04 | -0.091 | 0.005 |
| **Hyperactivity Scale** | | | | | |
| Restless | -0.113* | 0.302* | 0.563* | 0.040* | 0.309* |
| Fidget | -0.021 | 0.260* | 0.552* | 0.038* | 0.327* |
| Attention | 0.093* | 0.077 | 0.737* | -0.102* | 0.046* |
| Task | 0.097* | -0.088* | 0.708* | -0.327* | -0.051* |
| **Peer Problems** | | | | | |
| Alone | 0.348* | -0.216* | 0.002 | -0.228* | 0.484* |
| Friend | 0.164* | 0.022 | 0.025 | -0.247* | 0.398* |
| Bullied | 0.427* | 0.188* | 0.035 | 0.017 | 0.301* |
| Adults | 0.187* | 0.015 | 0.007 | -0.028 | 0.573* |
| **Pro-Social Scale** | | | | | |
| Considerate | 0.017 | -0.378* | 0.033 | 0.511* | -0.036 |
| Shares | -0.015 | -0.228* | 0.053* | 0.569* | -0.074* |
| Helpful | 0.033 | -0.048 | 0.053* | 0.771* | -0.02 |
| Kind | 0.034 | -0.120* | -0.021 | 0.653* | -0.034 |
| Volunteers | -0.002 | -0.005 | -0.114* | 0.677* | 0.223* |

*.*

***Table S12 Footnote:*** *Geomin Rotated Loadings for Five-Factor Model with items “impulse” and “liked” removed.* ** Indicates p<0.05. Loadings greater or equal to 0.4 are highlighted in dark grey. Greater or equal to 0.35 are highlighted in light grey*

**Table S13 - Model Fit Indices for Factorial Structure with Cross-Loadings Removed**

| **Model** | **χ2** | **df** | **CFI** | **TLI** | **RMSEA** | **SRMR** |
| --- | --- | --- | --- | --- | --- | --- |
| 7) Baseline five-factor Model with cross-loadings removed | 1567.287 | 220 | 0.939 | 0.930 | 0.033 | 0.056 |
| 8) Five-factor model with correlations between unique variances and cross-loadings removed | 1191.873 | 213 | 0.956 | 0.948 | 0.028 | 0.051 |
| 9) Baseline three-factor Model with cross-loadings removed | 2337.829 | 227 | 0.905 | 0.894 | 0.040 | 0.070 |
| 10) Three-factor model with correlations between unique variances and cross-loadings removed | 1497.401 | 220 | 0.942 | 0.934 | 0.032 | 0.057 |
| 11) Second-order two-factor model with cross-loadings removed | 1624.597 | 223 | 0.937 | 0.928 | 0.033 | 0.058 |
| 12) Second-order two-factor model with correlations between unique variances and cross-loadings removed | 1260.741 | 216 | 0.953 | 0.945 | 0.029 | 0.053 |

***Table S13 Footnote:*** *Sensitivity analysis showing model fit for competing models with cross-loading items removed (“impulse” and “liked”). Abbreviations: χ2 – chi-squared; df – degrees of freedom; CFI – Comparative Fit Index; TLI – Tucker-Lewis Index; RMSEA – Root Mean Square Error of Approximation; SRMR – Standardized Root Mean Squared Residual.*

**Table S14- Average Variance Explained for Factorial Structure with Cross-Loadings Removed (Sensitivity Analysis): First-order five-factor model**

|  |  | **Correlation** | | | | | **Squared Correlation** | | | | |
| --- | --- | --- | --- | --- | --- | --- | --- | --- | --- | --- | --- |
|  | **Ave** | **Peer** | **Emotion** | **Conduct** | **Hyper** | **Social** | **Peer** | **Emotion** | **Conduct** | **Hyper** | **Social** |
| **Peer** | 0.45 |  |  |  |  |  |  |  |  |  |  |
| **Emotion** | 0.52 | 0.85 |  |  |  |  | 0.72 |  |  |  |  |
| **Conduct** | 0.54 | 0.60 | 0.64 |  |  |  | 0.36 | 0.40 |  |  |  |
| **Hyper** | 0.41 | 0.59 | 0.63 | 0.79 |  |  | 0.35 | 0.40 | 0.62 |  |  |
| **Social** | 0.49 | -0.41 | -0.34 | -0.70 | -0.51 |  | 0.17 | 0.12 | 0.49 | 0.26 |  |

***Table S14 Footnote:*** *Sensitivity analysis showing AVE scores for first-order factors with cross-loading items removed (“impulse” and “liked”). AVE – Average Variance Explained.* *Shorthand name “Peer” refers to Peer Problems, “Emotion” to Emotional Symptoms, “Conduct” to Conduct Problems and “Hyper” to Hyperactivity.*

**Table S15 - Average Variance Explained for Factorial Structure with Cross-Loadings Removed (Sensitivity Analysis): Second-order factor model**

|  |  | **Correlation** | | | **Squared Correlation** | | |
| --- | --- | --- | --- | --- | --- | --- | --- |
|  | **Ave** | **Intern** | **Extern** | **Social** | **Intern** | **Extern** | **Social** |
| **Intern** | 0.85 |  |  |  |  |  |  |
| **Extern** | 0.79 | 0.76 |  |  | 0.57 |  |  |
| **Social** | 0.49 | -0.40 | -0.68 |  | 0.16 | 0.47 |  |

***Table S15 Footnote:*** *Sensitivity analysis showing AVE scores for second-order factors with cross-loading items removed (“impulse” and “liked”). AVE – Average Variance Explained. Shorthand name “intern” refers to internalising symptoms, “extern” to externalising symptoms, “social” to pro-social scale.*

**Table S16 - Sensitivity Analysis of Predictive Validity for Factorial Structure with Cross-Loadings Removed: First-order five-factor model**

|  | **Mutually Adjusted** | | | **Minimally Adjusted** | | |
| --- | --- | --- | --- | --- | --- | --- |
|  | Estimate | SE | P Value | Estimate | SE | P Value |
| **Depression** | | | | | | |
| Emotion | 0.41 | 0.11 | <0.001 | 0.35 | 0.09 | <0.001 |
| Peer | -0.11 | 0.08 | 0.142 | -0.09 | 0.07 | 0.225 |
| Conduct | 0.38 | 0.16 | 0.016 | 0.31 | 0.13 | 0.019 |
| Hyper | -0.24 | 0.1 | 0.012 | -0.16 | 0.08 | 0.044 |
| Social | 0.14 | 0.09 | 0.124 | 0.08 | 0.08 | 0.303 |
| **ADHD** | | | | | | |
| Emotion | -0.57 | 0.18 | 0.001 | -1.08 | 0.36 | 0.003 |
| Peer | 0.53 | 0.18 | 0.003 | 0.89 | 0.28 | 0.002 |
| Conduct | 0.17 | 0.14 | 0.242 | 0.27 | 0.2 | 0.181 |
| Hyper | 0.59 | 0.1 | <0.001 | 0.76 | 0.14 | <0.001 |
| Social | 0.01 | 0.09 | 0.907 | 0.05 | 0.12 | 0.708 |
| **Autism** | | | | | | |
| Emotion | -0.42 | 0.16 | 0.009 | -0.41 | 0.17 | 0.016 |
| Peer | 0.94 | 0.14 | <0.001 | 0.91 | 0.22 | <0.001 |
| Conduct | -0.45 | 0.18 | 0.011 | -0.43 | 0.2 | 0.03 |
| Hyper | 0.53 | 0.12 | <0.001 | 0.5 | 0.16 | 0.001 |
| Social | -0.32 | 0.09 | <0.001 | -0.3 | 0.11 | 0.005 |

***Table S16 Footnote:*** *Sensitivity analysis showing regression coefficients for probit regression between first-order factors with cross-loading items removed (“impulse” and “liked”) and clinical outcomes at age 14 (ADHD and Autism) and 13 to 17 (Depression).*

**Table S17 - Sensitivity Analysis of Predictive Validity for Factorial Structure with Cross-Loadings Removed: Second-Order Factor Model**

|  | **Mutually Adjusted** | | | **Minimally Adjusted** | | |
| --- | --- | --- | --- | --- | --- | --- |
|  | Estimate | SE | P Value | Estimate | SE | P Value |
| **Depression** | | | | | | |
| Internalising | 0.37 | 0.09 | <0.001 | 0.31 | 0.08 | <0.001 |
| Externalising | 0.01 | 0.11 | 0.93 | 0.06 | 0.1 | 0.56 |
| Pro-social | 0.06 | 0.07 | 0.4 | 0.01 | 0.06 | 0.88 |
| **ADHD** | | | | | | |
| Internalising | -0.23 | 0.1 | 0.02 | -0.15 | 0.09 | 0.09 |
| Externalising | 0.96 | 0.12 | <0.001 | 0.84 | 0.11 | <0.001 |
| Pro-social | 0.14 | 0.08 | 0.09 | 0.14 | 0.08 | 0.06 |
| **Autism** | | | | | | |
| Internalising | 0.55 | 0.07 | <0.001 | 0.58 | 0.07 | <0.001 |
| Externalising | 0.04 | 0.11 | 0.68 | -0.01 | 0.1 | 0.95 |
| Pro-social | -0.25 | 0.08 | 0.001 | -0.22 | 0.07 | <0.001 |

***Table S17 Footnote:*** *Sensitivity analysis showing regression coefficients for probit regression between second-order factors with cross-loading items removed (“impulse” and “liked”) and clinical outcomes at age 14 (ADHD and Autism) and 13 to 17 (Depression).*
